# Are Tau Aggregates Toxic or Protective in Tauopathies?

**DOI:** 10.3389/fneur.2013.00114

**Published:** 2013-08-13

**Authors:** Catherine M. Cowan, Amrit Mudher

**Affiliations:** ^1^Centre for Biological Sciences, University of Southampton, Southampton, UK

**Keywords:** Alzheimer’s disease, dimer, oligomer, filament, neurofibrillary tangle, insoluble tau

## Abstract

Aggregation of highly phosphorylated tau into aggregated forms such as filaments and neurofibrillary tangles is one of the defining pathological hallmarks of Alzheimer’s disease and other tauopathies. Hence therapeutic strategies have focused on inhibition of tau phosphorylation or disruption of aggregation. However, animal models imply that tau-mediated dysfunction and toxicity do not require aggregation but instead are caused by soluble hyper-phosphorylated tau. Over the years, our findings from a *Drosophila* model of tauopathy have reinforced this. We have shown that highly phosphorylated wild-type human tau causes behavioral deficits resulting from synaptic dysfunction, axonal transport disruption, and cytoskeletal destabilization *in vivo*. These deficits are evident in the absence of neuronal death or filament/tangle formation. Unsurprisingly, both pharmacological and genetic inhibition of GSK-3β rescue these tau phenotypes. However, GSK-3β inhibition also unexpectedly increases tau protein levels, and produces insoluble granular tau oligomers. As well as underlining the growing consensus that tau toxicity is mediated by a highly phosphorylated soluble tau species, our findings further show that not all insoluble tau aggregates are toxic. Some tau aggregates, in particular tau oligomers, are non-toxic, and may even be protective against tau toxicity *in vivo*. This has serious implications for emerging therapeutic strategies to dissolve tau aggregates, which might be ineffective or even counter-productive. In light of this, it is imperative to identify the key toxic tau species and to understand how it mediates dysfunction and degeneration so that the effective disease-modifying therapies can be developed.

## Introduction

### Tau protein in Alzheimer’s disease and other tauopathies

Deposits of insoluble tau within neurons are defining pathological hallmarks in the group of neurodegenerative diseases known as tauopathies. Tauopathies include Alzheimer’s disease (AD), Fronto-temporal Dementia with Parkinsonism on chromosome-17 (FTDP-17), Pick’s disease, Corticobasal Degeneration (CBD), Progressive Supranuclear Palsy (PSP), and others (1). In all of these conditions, tau becomes both abnormally hyper-phosphorylated and deposited in insoluble aggregates [reviewed in Ref. (1, 2)]. These diseases differ in their clinical features, differentially-affected neuronal populations, and the distinct forms taken by the insoluble tau. Indeed, even within one disease state, the insoluble tau may be found in many distinct morphological forms; some *en route* to the final form of that disease’s tau deposits, and others possibly on a different pathway.

In this review we will focus primarily on the forms of insoluble tau observed in AD, since they have been more widely studied. We will describe the different species of insoluble tau that have been identified; briefly review the factors that might promote tau aggregation; and then assess the evidence for and against the toxicity of each type of tau aggregate. Inevitably, this cannot be a comprehensive account of the extensive literature on this subject in the interests of space. Therefore we have selected papers which we believe represent the balance of evidence for and against toxicity, with apologies to those whose work we have not included. In this context we will use the term toxicity rather broadly, meaning either neuronal death, or neuronal dysfunction without death.

## Physiological and Pathological Species of Tau

This section briefly describes the major forms that tau has been shown to take in AD. These different species are treated in approximate order of size, from smallest to largest (Table 1). However, there is no intention to imply that each one goes on to form the next in a clear pathway.

**Table 1:** **Summary of the major forms of tau identified**.

Species of tau	Abnormally phosphorylated?	Toxic?
Monomer	Sometimes	Probably only when aberrantly phosphorylated
Dimer/trimer	Sometimes	Some types shown to be sufficient for toxicity
Small soluble oligomer	Sometimes	Some types shown to be sufficient for toxicity
Granular Tau oligomer	Sometimes	Not always
Filament	Yes	Might comprise of toxic tau, yet filaments themselves are probably neither necessary nor sufficient for toxicity
Neurofibrillary tangle	Yes	Might comprise of toxic tau, yet tangles themselves are probably neither necessary nor sufficient for toxicity
Ghost tangle	Yes	Unlikely

### Monomer

Monomers of tau are highly soluble proteins of 55–74 kDa in size [depending upon splice variant and phosphorylation status – (3)]. There are six splice variants which contain either three or four microtubule-binding repeats, as well as either zero, one, or two N-terminal domains. These isoforms are usually denoted tau^0N3R^, tau^1N3R^, tau^2N3R^, tau^0N4R^, tau^1N4R^, and tau^2N4R^. They usually acquire a predominantly random coil structure under normal physiological conditions (4). Partially folded forms of tau monomers have also been described which are distinct from native tau monomers, and have a reduced level of random coiling but an increased level of β-sheet structure (5). Interestingly, such molecules are immediately positive for Thioflavin (which binds β-sheet). Compact monomers have also been characterized displaying intra-molecular disulfide bonds (6). Only the three isoforms of four-repeat tau can form these compact monomers, since the second cysteine required for an intra-molecular interaction is in the extra repeat domain.

### Dimer/trimer

Dimers are composed of two tau monomers in anti-parallel orientation linked by disulfide bonds. Tau dimers can be observed by electron microscopy (EM) as rod-like particles 22–25 nm long, which is similar in appearance to the monomers (7). Dimers can form from any isoforms of tau. Within that, however, two distinctly different forms of dimers have been described (8). One is cysteine-dependent and reducible; while in contrast the other is cysteine-independent, non-reducible, and has inter-molecular disulfide bridging at the microtubule-binding domain. Both forms have been identified *in vitro*, and in tau transgenic (JNPL3) mice (8). Preparing small oligomers from recombinant tau *in vitro*, dimers have been reported with apparent sizes of 180 kDa (9) and 130 kDa (10), as well as trimers with an apparent size of 120 kDa (11). In human tau transgenic mice, soluble tau species of 140 kDa have been described (8, 12). Small soluble tau species of approximately dimer and trimer size, and probably including tau fragments, have also been isolated from synapses in AD brains (13). It is unclear whether these variously reported dimers and trimers are indeed different tau species or whether they represent subtle variations of the same structure.

### Small soluble oligomer

Small soluble oligomers of tau of very many different sizes have been described *in vitro* and *in vivo*. Often, however (perhaps because of differences in post-translational modifications leading to different apparent sizes on PAGE), it can be difficult to determine if small oligomers described by different groups represent the same species or not. In one study, the soluble dimers described above were shown *in vitro* to develop into small soluble oligomers containing six to eight tau molecules (approximately 300–500 kDa in size) (8). JNPL3 mice, which over-express human tau with the P301L mutation (tau^0N4R-P301L^) and harbor neurofibrillary tangles (NFTs), additionally have small tau oligomers which run at a wide range of sizes by PAGE [Sahara et al. (8)].

### Insoluble granular tau oligomer

Granular tau oligomers (GTOs) are electron-dense granular or globular aggregates of tau. They have been isolated from AD brains, mostly at early and moderate Braak stages (14). GTOs are composed of an average of 40 densely packed tau monomers. This corresponds to a size of 1800 kDa, or 20–50 nm in diameter when observed by EM or by atomic force microscopy (AFM) (15). It is important to note that, on the scale of insoluble protein aggregates generally, this is extremely small. Standard protocols for the sedimentation of insoluble proteins, such as 100,000 × *g* spin for 30–60 min [e.g., Ref. (16)], would fail to sediment GTOs which would remain in suspension in the “soluble” fraction, despite their demonstrable insolubility in SDS (15). Instead, sedimentation of GTOs requires a 200,000 × *g* spin for 2 h (15). The same authors developed a rigorous fractionation/purification protocol for GTOs. They further characterized the GTOs as being positive for MC1 and for Thioflavin, despite clearly being not filamentous in any way. They conclude that GTOs have β-sheet structure, and suggest that they may be composed of the partially folded form of tau monomer (15).

### Filament

It is well known that tau is capable of polymerization into filamentous forms. In AD, the predominant filaments are Paired Helical Filaments (PHF) and Straight Filaments (SF). In other tauopathies such as FTDP-17, however, there is variability in the morphology of tau filaments depending upon the tau mutations and/or tau isoforms involved. Here, filaments may take on other shapes such as twisted ribbon-like and rope-like filaments (17). A straight filament strand is 10 nm wide, and thus PHFs display alternating widths of 10 and 20 nm, with a half-periodicity of 80 nm (18, 19). Tau filaments exhibit β-sheet structure (20) which forms through the MT-binding repeat region (7, 21). Tau filaments from human AD brain have been shown to contain all six tau isoforms (22), although *in vitro* they can also be formed from single isoforms. They can be considered an amyloid (23, 24).

### Pretangle

The pretangle is a slightly confusing concept that historically may have referred to a variety of species of tau, or even the status of a neuron. Generally speaking, a pretangle neuron is one that is positive for abnormal tau epitopes (misfolded and/or hyper-phosphorylated), in some insoluble format large enough to be visible by light microscopy, yet free from mature fibrils or tangles by morphology. Bancher et al. (25) helpfully classified tangles into four stages (0–3). In this system ^1^, stage 0 tangles (later referred to by others as pretangles) are identified by cytoplasmic non-fibrillar (granular or diffuse) tau immunoreactivity, visible at the light microscope level. When viewed by EM, the labeled material was found to consist of PHFs, SFs, and smaller granular electron-dense material. Where pretangles were observed as granular via light microscopy, this probably represents non-filamentous clumps of PHFs, SFs, and the ultrastructural granules. Other researchers have described immunoreactivity for certain abnormal AD-associated tau epitopes in neurons containing no fibrils, and have deemed the neurons so labeled to be at a pretangle stage [for example Alz50 (26), the 12E8 epitope S262/S356 (27, 28), and T231 (27, 29)]. Confusingly, there are a number of conflicting reports in the literature as to whether “pretangles” are silver-staining, thioflavin-positive, and whether or not they contain β-sheet structure. It seems probable that these discrepancies arise from (a) a heterogeneity of what is meant by “pretangle” and (b) a sensitivity issue in regard to the assays for β-sheet. Pretangles should surely be positive for markers of β-sheet, since even the earliest partially-folded monomer (5) and certainly tau filaments (30) demonstrably contain β-sheet structure.

### Other large non-fibrillar tau aggregates

There are other forms and morphologies of pathological insoluble tau found in human brains which are large enough to be seen with the light microscope, and may be filamentous, yet are non-fibrillar in structure. Such aggregates include Hirano bodies, Pick bodies, and argyrophilic grains.

Hirano bodies have been described in AD, Pick’s disease and other tauopathy brains (31, 32). Hirano bodies are large intraneuronal paracrystalline structures of 5–10 μm in width by 10–30 μm in length, composed of 7 nm filament arrays (32). They contain tau, other microtubule-associated proteins, actin, cofilin, other actin-binding proteins, and a fragment of APP.

Pick bodies are the characteristic morphology assumed by tau filaments in Pick’s disease, in which they accumulate in limbic and cortical neurons. They are large structures that vary in size in different neuronal types, but are approximately the size of the nucleus. Pick bodies are formed of disorganized bundles of filaments which comprise only the three 3-repeat isoforms of tau, in contrast to the PHFs and SFs formed in AD which are made of all six isoforms [reviewed in Ref. (33)].

Argyrophilic grains are found in Argyrophilic Grain disease, where they accumulate in both neuronal processes and oligodendrocytes (34). Argyrophilic grains are structures that may be spherical, oval, comma-shaped, or spindle-shaped. As the name suggests, they are readily detectable by conventional silver-staining and light microscopy. The grains are much smaller than Hirano bodies, Pick bodies, and NFTs at approximately 4–9 μm in size. Argyrophilic grains are comprised of four-repeat tau in 9–18 nm SF and bundles of 25 nm smooth tubules. They never contain PHFs and ribbon-like filaments (34–36).

### Neurofibrillary tangle

Neurofibrillary tangles are the classic tangles first described by Alzheimer in 1907. Classified by Bancher et al. (25) as stage 2 tangles and often described as “flame-shaped,” they are large bundles of fibers consisting of both PHFs and SFs which may fill the entire neuronal cytoplasm. The fibers are silver-staining. Brief mention should be made here also of neuropil threads, which are bundles of SFs and PHFs occupying dendrites and largely displacing the cytoskeleton (37).

### Ghost tangle

Ghost tangles are the structures that remain when the neuron within which the tangle formed has degenerated. They comprise large extracellular bundles of loosely arranged tau filaments. Compared to NFTs, ghost tangles stain more weakly for tau and more strongly for ubiquitin (25). It is thought that ghost tangles have undergone substantial proteolysis, and that thus the filaments are comprised predominantly of tau fragments, again in contrast to NFTs (38).

## The Sequence of Events in Tau Aggregation

**Figure 1:**
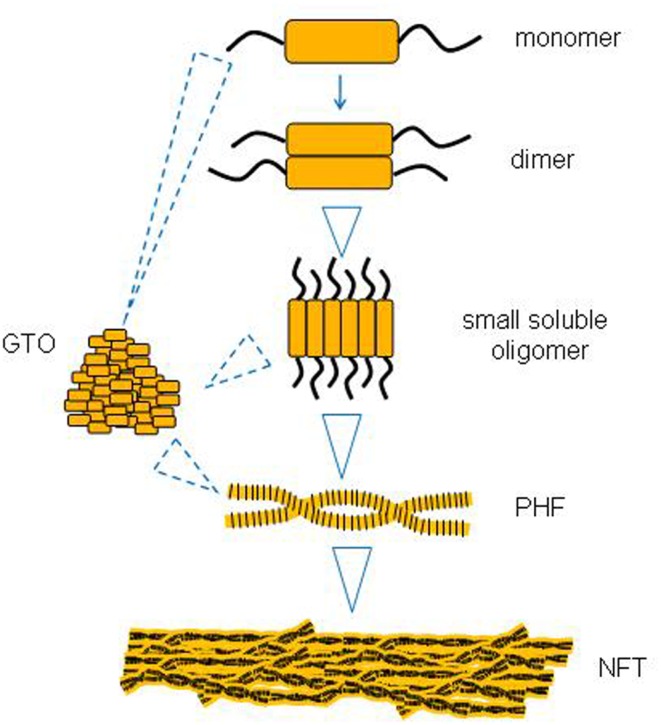
**A putative sequence of events in tau aggregation into neurofibrillary tangles (NFTs)**.

There is some evidence to suggest that larger tau aggregates like PHFs and NFTs evolve from the successive aggregation of smaller tau species like monomers and soluble oligomers (Figure 1). One missing link appears to be whether small oligomers can form directly into GTOs in a linear pathway, or whether they represent two different pathways from monomers to PHFs and NFTs.

### Monomer → dimer

There is evidence from the kinetics of tau polymerization that, once the partially folded conformation of the monomer has formed (however that may be triggered), then the process from monomers to dimers (and thence to oligomers) is energetically favorable and proceeds spontaneously (5). For monomers to be able to form dimers requires the PHF6 hexapeptide in the third microtubule-binding repeat domain (8, 39). However, the compact form of the tau monomer does not participate in this form of aggregation (40).

### Dimer → small soluble oligomer

The tau dimer, in particular the cysteine-independent, non-reducible form (8), is thought to be an important intermediate which is involved in controlling the rate of formation of larger intermediates and fibrillization (6, 7, 41). In addition, more than one group has demonstrated that *in vitro* generated tau dimers aggregate to form larger tau oligomers (8, 9).

### Small soluble oligomer → GTO

We are not aware of any direct evidence that small oligomers proceed to form GTOs. There is, however, evidence that tau monomers *in vitro* can form GTOs (15), as well as that both monomers and GTOs can form PHFs. However, whether the sequence always proceeds from monomer → dimer → small oligomer → GTO → filament, or whether GTOs and other types of tau oligomers can be on different pathways, is not clear. In general, it is believed that when tau forms larger structures such as filaments of differing morphology, the interactions between tau molecules remain the same, and subunit packing follows the same plan (40). On these grounds it is plausible that GTOs might be part of the same pathway.

### GTO → PHF

Increasing the concentration of GTOs *in vitro* causes them to form filaments, whereas the constituent soluble tau does not. On the basis of this, it is suggested that GTOs are precursors of PHFs (15).

### Monomer/dimer → PHF (possibly via the other intermediates)

There is a wide variety of evidence showing that monomers can polymerizes into PHF, but that does not address whether this is via the oligomeric intermediates or not. Such evidence includes the early *in vitro* demonstrations that tau at high concentrations will self-assemble into PHFs (42–44), and evidence that dimers are normally rate-limiting intermediates in this process *in vitro* (4, 6). There followed from these studies a large body of work delineating important details such as which motifs within tau are required for fibrillization, in which monomeric tau clearly formed into PHFs (39, 45–47). However, as in the early studies, whether oligomers were formed on the way was not necessarily assessed directly.

The mechanism for PHF formation requires two hexapeptide motifs in the microtubule-binding region of tau. These are PHF6 [(306)VQIVYK(311)] and PHF6^∗^ [(275)VQIINK(281)]. Formation of PHFs involves these two motifs changing conformation from random coil to β-sheet structure (24, 39). It should be noted that mutant tau containing no cysteines is still able to form PHFs *in vitro*, even though much more slowly than WT tau (40). This means that cysteine-dependent (covalent) dimers are not a requisite stage between monomer and PHF.

### PHF → NFT

It is well established that NFTs *in vivo* are composed of PHFs and SFs (25). Furthermore, there is also direct *in vitro* evidence that filaments will spontaneously clump together into NFTs (48). Thus it is highly likely that NFTs are formed by the accumulation of tau filaments.

## What Promotes Tau Aggregation?

Little is known about what first triggers the initiation of tau aggregation. It is known that normal monomers do not spontaneously seed aggregation, and that some sort of trigger is needed (30, 49). However, numerous factors have been identified that can promote or increase tau aggregation, at least *in vitro* [reviewed in Ref. (50)].

Enzymatic cleavage of the tau monomer is one such factor. Truncation of the tau protein at Glu391 (51, 52), truncation by caspases at Asp421 (53), cleavage by thrombin (54), removal of the C terminus of the protein (55, 56), or deamination at asparagine or glutamine residues (57) have all been shown to promote tau aggregation [Reviewed in Ref. (58)].

Local concentration of tau can be key. Tau at high concentrations *in vitro* forms PHFs (42–44). Moreover, the transition of tau from random coil to β-sheet is also known to be concentration dependent (39), further supporting the idea that excessive local accumulation of tau may promote its aggregation (especially if other pro-aggregating factors – such as those discussed below – are also in the near vicinity).

Controversially, tau phosphorylation has been postulated to both stimulate and repress its subsequent aggregation into filaments. Circumstantial evidence for stimulation includes the seminal fact that filamentous tau is highly hyper-phosphorylated (59) at many sites. More direct but *in vitro* evidence shows that tau phosphorylated at AD sites polymerizes more readily into tangles of PHF/SF; dephosphorylation abolishes tau’s self-assembly; and hyperphosphorylation of recombinant tau by brain kinases induces its self-assembly into tangles of PHF/SF (60, 61). In a cellular model, it has been shown that all three kinases GSK-3β, MEKK, and JNK3 are required for tau aggregation (62). Phosphorylation of tau specifically at Thr231, Ser396, Ser422, and Ser404 promotes self-aggregation of tau into filaments (55, 63, 64). *In vivo*, overexpression of the kinases GSK-3β or Cdk5 can promote tau aggregation (65, 66). On the other hand, *in vitro* studies have shown that tau phosphorylation is not necessary to drive tau into PHFs (41, 67). On the contrary, phosphorylation of KXGS motifs in the repeat region *inhibits* tau aggregation *in vitro* (54, 68). Furthermore, more recently emerging data showing that tau aggregates made up of recombinant non-phosphorylated tau can “seed” further tau aggregation in cells (discussed below) also supports the idea that phosphorylation is not required to promote aggregation (69, 70). However, it is not yet known whether phosphorylated tau would seed and promote aggregation at a different rate.

Some of the missense and deletion mutations found in tau in cases of fronto-temporal dementia (FTDP-17), when expressed in models, display enhanced aggregation compared to normal tau. *In vitro* PHF formation is faster for recombinant tau harboring one of various such mutations. Human tau with each one of the point mutations G272V, N279K, V337M, or R406W shows significantly faster *in vitro* PHF formation than WT full-length human tau, while the ΔK280 and P301L mutants form PHFs at dramatically greater speeds (46). This phenomenon has been confirmed *in vivo*: mice expressing mutant human tau^P301L^ develop pathology more readily than those expressing WT human tau, both on 0N4R and 2N4R tau backgrounds (71–73).

Many polyanionic cofactors of all kinds can promote PHF assembly. These include glycosaminoglycans (GAGs) such as heparin and neuroparin (40, 74, 75); sulphoglycosaminoglycans (sGAGs) like keratins or chondroitin sulfates (76), RNA (41); polyglutamic acid (30, 41, 74); fatty acids such as arachadonic acid (77, 78) and alkyl sulfate detergents (79).

Other factors which may promote tau aggregation include tissue transglutaminase (80), Congo red (81), ferritin (82), H_2_O_2_ in the presence of iron (Fenton’s reaction) (83), and quinones (84).

Despite this wealth of data over many years regarding factors that promote aggregation, questions still remain about what initiates tau aggregation *in vivo* in health and disease. However, once tau aggregation has been initiated, it is believed to promote further “prion”-like “seeding” and propagation of tau aggregation and pathology (85). This was first demonstrated *in vivo* where stereotaxic injections of brain homogenate containing tau aggregates led to induction and propagation of tau aggregation in tau transgenic mice (85). Supportive data also emerged from studies in cell culture showing that incubation of tau-expressing cells with fibrils of recombinant tau leads to induction of tau aggregation in the recipient cells (69, 70).

## Are Neurofibrillary Tangles Toxic?

### NFTs: Evidence for toxicity

The evidence that associates NFTs with neuronal dysfunction and neurodegeneration is largely correlative in nature. Studies of human post-mortem brains initially implicated NFTs in toxicity by showing a strong spatial and temporal correlation between NFTs and severity of dementia, and between NFTs and neurodegeneration or neuronal death (86–92). Some tau transgenic mouse models display neuron loss in the same timeframe and/or location as NFT formation. For example, expression of tau^P301L^ under the thy1.2 promoter causes neuronal apoptosis at the same age as filaments and NFTs (93); while tau^P301L^ under the prion promoter causes NFTs in spinal cord, brainstem, and pretangles in cortex, at the same time as loss of motor neurons (71). Furthermore, in tau mouse models, there is a correlation between reduction of NFT and improvement in cognition (94). The limitation of these correlative studies between reduced NFTs and reduced impairments is that, in many cases, other smaller tau species may be reduced also. This leaves open the question of whether it is the reduction of the NFTs or the smaller species which has been beneficial.

More direct evidence in favor of NFT toxicity comes from mice conditionally expressing human tau fragments harboring pro-aggregation or anti-aggregation mutations (95, 96). Pro-aggregation mice develop PHFs, “pretangles,” and NFTs early; followed by synaptic and neuronal loss. In constrast, mice expressing the same tau molecule but with the anti-aggregation mutation never develop aggregates or neuronal pathology. This was verified in a *C. elegans* model of tauopathy which went further to show that treatment with anti-aggregation inhibitors protected against tau-mediated toxicity (97). This suggests that tau aggregation in the form of PHFs or larger has been the cause of cell death. Moreover, when expression of human tau is suppressed, mice are rescued from toxicity in terms of both cell death and cognition. This represents very strong evidence in favor or PHFs and/or NFTs as the toxic species. These conclusions were difficult to reconcile with a body of evidence detailed below proving that PHFs and NFTs are neither necessary nor sufficient for toxicity. However, the authors subsequently showed that in the animals expressing pro-aggregant tau, toxicity might in fact be mediated by a species of tau smaller than PHFs and NFTs (98, 99).

### NFTs: Evidence against toxicity

There is now strong evidence from a number of models that NFTs are not required for tau-induced neuronal dysfunction and toxicity. In most *Drosophila* models of tauopathy, neuronal NFTs are usually not formed at all, despite clear neurodegeneration, and functional phenotypes (100–104). In some mouse models where NFTs do form, cognitive/behavioral impairments and cell death can be demonstrated *earlier* in the time course of the disease prior to NFT formation (105). In a different mouse model [PrP44: the shortest human tau isoform (tau^0N3R^) under the prion promoter] the formation of tau filaments coincides in the time course of the disease with phenotypes such as neurodegeneration and motor deficits, while NFTs form later (106, 107).

There is also compelling evidence that NFTs are not sufficient for toxicity, from mice that conditionally express human tau (tau^0N4R-P301L^). These mice display age-dependent development of NFTs, neuronal loss, and progressive motor deficits. When tau expression is switched off after the onset of memory impairments and NFT formation, memory improves and cell loss is stabilized, yet NFTs remain (108, 109). Furthermore, when tau is turned off at a timepoint when there are pretangles but no NFTs yet, the pretangles stay stable. This indicates that they also are insufficient for toxicity. This is corroborated in a different study using the same mice, in which a successful treatment reduced motor deficits despite failing to reduce NFTs (110). Furthermore, tangle-bearing neurons in this model were shown to be just as active in a functional hippocampal circuit as non-tangle bearing neurons (as evidenced by expression of the immediate early gene *Arc* in response to environmental cues) (111). Further investigations of the mouse conditionally expressing pro- and anti-aggregant form of tau, mentioned in the preceding section, also supports this view. The pro-aggregation mice develop learning and memory deficits from which they recover after tau expression is turned off (98). However, it transpires that after an extended period of tau suppression, NFTs still remain, as in the tau^0N4R-P301L^ mice. This implies that it is a smaller species of tau (soluble or insoluble) which has decreased in correlation with behavioral improvement in these studies (98).

In the light of such evidence, it has been suggested that formation of NFTs is a protective response that ultimately fails (58). This review describes a scenario in which caspases, having become activated because the cell contains toxic tau and is thus under stress, cleave the tau making it more fibrillogenic. Cleavage is unlikely to be the only trigger, since the initial steps of aggregation can involve primarily full-length tau isoforms (5, 112). Either way, the idea is that once tau aggregates seed, they can sequester toxic tau and thus delay cell death. However, the trade-off is that axonal transport is compromised and cellular protein degradation pathways become clogged, and thus the neuron gradually becomes dysfunctional (58). This is supported by evidence that NFT-bearing neurons appear to survive for decades (113) and maintain markers of normal gene expression (111), and may be in fact be longer lived than those neurons without NFTs in the AD brain that would have died at earlier time-points and hence were not evident at post-mortem.

In conclusion, despite NFTs being a vital historical clue to the involvement of tau in neurodegenerative disease, a wide variety of strong evidence now exists that NFTs themselves are neither necessary nor sufficient to cause tau-induced toxicity and dysfunction.

## Are Tau Filaments Toxic?

Some of the evidence regarding NFTs is also applicable as indirect evidence about tau filament toxicity. That is, some of the evidence in favor of large aggregates being toxic could really apply to either NFTs or PHFs/SFs or both. Further, the evidence that NFTs are not toxic naturally casts suspicion onto smaller pathological species such as filaments. However, direct evidence for PHFs as the primary toxic species, rather than something smaller, is sparse.

### Filaments: Evidence for toxicity

As with the evidence in favor of NFT toxicity described above, much of the evidence that implicates filaments as a toxic species of tau is correlative in nature. For example, in some mouse models of tauopathy, filaments coincide in the time course of the disease with phenotypes such as neurodegeneration and motor deficits (106), while NFTs form later (107). Similar results were seen in one *Drosophila* model of tauopathy in which tau filament formation was reported (114). Furthermore, mutations in tau which are responsible for FTDP-17 also promote faster tau filament formation (46), thus circumstantially implicating tau filaments in the disease process.

An immunotherapy study targeting tau in a mouse model provided some more direct evidence in favor of PHF toxicity. In this study, immunization of JNPL3 mice (with a short phosphorylated tau fragment) served to reduce tau aggregation (into PHFs or larger aggregates) and the associated behavioral phenotypes, but failed to reduce smaller species of tau. This suggests that that PHFs or larger tau aggregates are the toxic species in this model (115). In another study, tau filaments but not monomers (at physiological concentrations) were shown to selectively impair anterograde transport in isolated squid axoplasm (116, 117).

### Filaments: Evidence against toxicity

As with the NFT situation, some animal models have impairment but no filaments, or at least not until later in the disease progression. For example, *Drosophila* (100–104) and *C. elegans* (118) expressing human tau display behavioral phenotypes indicative of neuronal dysfunction and toxicity without forming tau filaments or larger aggregates. Even in mice, in the transgenic tau model expressing the longest human tau isoform (tau^2N4R^), brains contain some form of insoluble tau but nothing as big as PHFs (or NFTs), while the animals display a motor impairment (119). Such evidence indicates that filaments are not necessary for tau-induced toxicity.

There is also evidence that filaments are not sufficient for toxicity, since they continue to form in the conditional tau mice mentioned earlier, in which transgenic tau expression has been turned off and deficits thereby rescued (108). In another mouse model, Andorfer et al. showed that, while there was widespread neurodegeneration, the PHF-containing neurons appeared “healthy” in terms of nuclear morphology, suggesting that the polymerized protein was probably neuroprotective (105). In an *in vivo* lamprey model (120), administration of a benzothiazole derivative drug (purported to break up tau filaments) successfully improved tau-induced phenotype, but apparently did so without actually breaking up the tau filaments. This provides further evidence that filaments are not sufficient for toxicity. *In vitro*, polymerization of hyper-phosphorylated tau into PHFs abolishes its toxic activity to sequester other MAPs (121). Unlike the soluble form of hyper-phosphorylated tau, the filamentous form of tau does not bind MAPs and does not disrupt microtubules *in vitro* (121).

In an inducible cell line, the repeat domain of wild-type tau was non-toxic, whereas a similar construct harboring a point mutation that induced aggregate formation (eventually PHFs) caused cell death (54). Crucially, however, increased cell death was observed *before* PHF formation in the aggregate-prone mutant, demonstrating that PHFs were not necessary for toxicity, and that in fact a smaller form of aggregate was the toxic species.

While the *in vivo* evidence is not quite as extensive as for NFTs, one can also conclude that tau filaments are neither necessary nor sufficient for tau-induced toxicity, and that something smaller than filaments is the most toxic form of tau.

### Species of tau found in filaments

When the insoluble protein fraction from brains of AD patients or animal models, containing any tau filaments or larger tau aggregates, is solublized using urea or formic acid, the tau species which were building blocks of these large insoluble aggregates can be identified. These species include monomers of 55, 60, 64, and 68 kDa in size (67), and a 170-kDa species. The 64 and 170 kDa species in particular have been implicated in toxicity. The 64-kDa species represents a hyper-phosphorylated monomer. It is found in brains of the tau^0N4R-P301L^ transgenic mouse (122), and increases with age in the insoluble fraction at the expense of the 55-kDa monomer (which is found in both soluble and insoluble fractions).

In the same study that showed NFTs were not necessary for toxicity [because they did not decrease in successfully treated tau mice – (110)], the successfully treated mice displayed a significant reduction in 64 kDa tau from the high-speed insoluble fraction. Confusingly, subsequent commentators have described this species as “soluble aggregated tau” [e.g., Ref. (123)]. However, this 64 kDa species is always a component of an aggregate that sediments at 150,000 × *g*, which is therefore bigger than a GTO. Another study in a conditional mouse model found that three distinct tau species correlated with neuronal dysfunction (12). Two of these species were in a sarkosyl-insoluble fraction (from which NFTs had been previously cleared) which must represent GTOs or filaments: a 64-kDa hyper-phosphorylated monomer and a 170-kDa hyper-phosphorylated oligomer. The third species was a 140-kDa oligomer from the soluble fraction. All of these species arose early in the disease progression, and increased with increasing learning and memory deficits. Conversely, all three decreased upon suppression of transgenic tau expression and recovery from neuronal dysfunction. These results clearly implicate one or more of the three species in toxicity; however, it is not clear whether the culprit is the soluble or insoluble components or both. The same 64 kDa species has also been described in a sarkosyl-insoluble fraction from transgenic tau^0N4R-301L^ mice that represents SFs and NFTs (71, 108). It is not toxic in this circumstance because it continues to increase after tau expression has been turned off and animals are recovering (108).

In summary, these two species of tau, a hyper-phosphorylated 64 kDa monomer and a 170-kDa oligomer, have frequently been demonstrated as components of an insoluble filament fraction. There is some evidence, although not conclusive, that these species may be associated with toxicity.

## Are (Insoluble) Granular Tau Oligomers Toxic?

Part of the difficulty in acquiring evidence about GTOs from the existing literature is that, as mentioned earlier, in standard insoluble protein fractionation protocols any GTOs in the sample will be lost. Even though fully insoluble, they are too small to sediment with a standard “high-speed insoluble” fraction (15). They remain in suspension in the “soluble” fraction; and yet as they are around 1800 kDa in size and not readily reducible without 8 M urea, they will not enter a standard PAGE resolving gel and be detected (Cowan, unpublished observation). Therefore when one reads, for example, studies in tau mouse models showing small oligomers and PHFs and drawing conclusions about toxicity, one cannot usually conclude anything about the presence or absence, toxicity or otherwise, of GTOs.

### GTOs: Evidence for toxicity

In the years following the identification of GTOs, when evidence was beginning to accumulate that PHFs and NFTs might not be toxic, some evidence remained that some insoluble form of tau must be toxic. There was speculation in reviews that insoluble oligomers of tau, perhaps GTOs, might be the most toxic species. However, there is no direct evidence for this. Clues that GTOs might be associated with toxicity come from studies showing that numbers of GTOs increase with progression of AD, that fewer GTOs are observed at Braak stage 0 than at stage 1, and that their peak precedes that of NFTs (14). GTOs are generally believed to consist of toxic phosphorylated species of tau because phosphorylated tau levels are high in the AD brain at the time-points when GTOs are abundant. Such clues have led to the suggestion that reducing GTOs may prove to be a promising therapeutic strategy; however, the authors of these publications acknowledge that the effect of GTOs on neuronal vulnerability is unknown.

There is also evidence that an insoluble oligomer(s) of some sort is probably the culprit, without clear evidence that it is GTOs. For example, in the study mentioned as evidence against PHF toxicity in an inducible cell line (54), the soluble tau construct was not toxic, whereas the aggregate-prone mutant tau species caused cell death *prior* to PHF formation. This therefore represents evidence against both soluble tau toxicity and PHF toxicity, but rather implicates some intermediate species. Similarly, in another study, very small insoluble tau oligomers (of up to a few hundred kDa) were isolated from synaptosomes derived from AD brains and were associated with impaired ubiquitin proteasome function (124). If any GTOs had been present in this preparation, they might not have been observed with the protocols used. Clearly small insoluble tau oligomers exist and are associated with toxicity but whether they can be classified as GTOs or indeed their precursors is not clear.

### GTOs: Evidence against toxicity

We have recently observed, in a *Drosophila* model of tauopathy, the formation of GTOs which are non-toxic (125) When flies express human tau^0N3R^ in neurons they exhibit a clear behavioral phenotype, but no insoluble tau. However, upon pharmacological or genetic manipulations which inhibit GSK-3β, the phenotype is rescued and GTOs are produced. We demonstrate that these GTOs produced in flies are the same size as those isolated from human brain and comprise of non-phosphorylated full-length tau monomers. Like us, another group has also demonstrated the formation of large insoluble tau oligomers in *Drosophila*, in conditions associated with rescue of tau-mediated toxicity (126). They showed that rescue of human tau^0N4R^ or tau^0N4R-R406W^ induced neurodegeneration and behavioral deficits by co-expression of Nicotinamide mononucleotide adenylyltransferase (NMNAT) also led to increased formation of insoluble tau oligomers. However whether these insoluble tau oligomers were the same as the GTOs that we described in our model is not clear. Nonetheless, both our studies collectively imply that tau aggregation can correlate with rescue from tau-induced phenotype. However, whether this is because of sequestration of smaller toxic tau species, or the presence of non-phosphorylated tau in the conditions in which the GTOs form, or something else about their structure is not clear.

Overall, the pathological significance of GTOs has yet to be fully understood. It is possible that different GTOs form in different circumstances and the phosphorylated status of their constituent tau proteins and/or the extent of β-sheet structure may play a role in determining their toxicity.

## Are Soluble (Monomeric or Oligomeric) Tau Species Toxic?

As has already been alluded to, there are numerous species of tau that are soluble, and it seems probable that they possess very different properties. Just considering monomers, there is clearly a multiplicity of species: the three major conformations described (regular, compact, and partially folded); the six splice variants; and of course the array of phosphorylation combinations, both demonstrated and possible, which have been barely touched upon here. Then there are at least two conformations of dimer (cysteine-dependent and independent), as well as trimers, and small oligomers of various sizes and phosphorylation states, as described earlier. We would speculate that there are probably many variations of soluble oligomeric tau species occurring in nature in the brain that have not yet been specifically described. Further, some of the evidence regarding the toxicity or otherwise of soluble tau cannot (or did not) differentiate between these species.

### Soluble tau: Evidence for toxicity

There are many examples of studies conducted *in vivo* and *in vitro* showing that soluble tau is sufficient to cause dysfunction and toxicity. To give a few examples, pseudophosphorylated tau causes cell death when virally expressed in hippocampal slices, without becoming SDS-insoluble (127). Soluble tau monomer applied extracellularly to cells in culture causes intracellular calcium increase and cell death (128, 129). Additionally, some tauopathy models in *Drosophila* (100–102, 130) exhibit significant neuronal dysfunction and degeneration and yet contain no insoluble tau providing strong evidence that soluble tau is sufficient to cause dysfunction and toxicity. Similar evidence exists in mouse models: for example, mice expressing WT tau^2N4R^ under the CamK-II promoter (131) as well as those expressing mutant tau^N279K^ (132) both display learning defects but no NFTs or insoluble tau and no cell death. However, these mouse studies are subject to the caveat discussed in Section “Are (Insoluble) Granular Tau Oligomers (GTOs) Toxic?” that if there were any GTOs present they would not have been detected by the protocols used. Therefore, strictly speaking, we feel that the conclusion from such studies is: either soluble tau or small insoluble tau oligomers are sufficient for toxicity.

Further evidence supporting soluble tau toxicity comes from many studies showing rescue of tau-mediated phenotypes after suppression of tau expression which leads to reductions in soluble tau but persistence of tangle pathology (58, 108, 111, 133). In one such study, transgenic tau^P301L^ mice were treated with methylene blue at a dose sufficient to rescue their memory deficit, and reduce total and soluble tau without affecting insoluble tau aggregates (134). This study went one step further in showing that the reduction of soluble tau is required for the rescue of phenotype.

Whether the toxic soluble species of tau is monomeric or oligomeric (or both) in these studies is not always clear. There is a convincing body of evidence showing that certain specific dimers, trimers, and other very small soluble oligomers are sufficient to cause toxicity both *in vitro* and *in vivo*. Patterson et al. (135) show that the 180-kDa dimers that they produced *in vitro* can suppress fast axonal transport in a squid axoplasm model. The 120-kDa trimers produced by Lasagna-Reeves et al. have been shown to be toxic *in vitro* (11) and *in vivo* (136). These trimers cause significantly more cell death than tau monomers or filaments when applied to SH-SY5Y cells in culture (11). Intra-hippocampal injections of trimers cause significant loss of synapses and neurons resulting in memory deficits, whereas injections of tau monomers or fibrils do not (136). Overall, these findings show that dimers and trimers of tau can be toxic.

Tau species of this size range have also been demonstrated *in vivo*, indicating that they are physiologically relevant. Both the Kayed and the Binder laboratories have used the oligomers that they made *in vitro* to raise oligomer-specific anti-tau antibodies, TOC-1 (9) and T22 (137), which they demonstrate recognize tau *in situ* in the post-mortem AD brain. Both oligomer-specific antibodies react with “pretangles” in early Braak stages, and co-localized with some disease-associated phospho-tau epitopes.

Small soluble oligomers also arise in many transgenic tau animal models in a context that implicates them in toxicity. For example, transgenic *Drosophila* expressing either human tau^0N4R^ or tau^0N4R-S406W^ in brain display soluble tau oligomers of 150–250 kDa in size (114, 126) and in both studies oligomer formation was associated with degeneration. Berger et al. (12) and Sahara et al. (8) independently identified small soluble tau oligomers of approximately 140 kDa (believed to be dimers) in brain homogenates of tau^0N4R-P301L^ transgenic mice. The oligomers detected by Berger et al. (12) (in the inducible tau^0N4R-P301L^ mice) appeared at very early stages of disease when memory deficits were evident in the absence of tangle formation or neuronal loss. Either this 140 kDa soluble species and/or the two small insoluble species of tau discussed in Section “Species of Tau Found in Filaments” are implicated in causing toxicity in this model.

Like the oligomers identified by the Binder and Kayed laboratories described above, the 140-kDa oligomers identified by Berger et al. (12) in tau transgenic mice are also detectable in the brains of AD and FTDP-17 patients. It is not clear whether all of these oligomers are one and the same tau multimer or whether they represent tau oligomers at different stages of maturation during the disease process. In addition, it has not been determined whether aggregation into larger oligomers alters the toxicity of the small tau oligomers.

### Soluble tau: Evidence against toxicity

Evidently, not all soluble phosphorylated tau can be toxic. Soluble tau of many species is obviously found physiologically in healthy individuals. One specific example of a soluble species known to be non-toxic is the compact monomer with intra-molecular disulfide bonds, which appears to be a species that is relatively protective and, notably, does not go on to form larger aggregates (40).

In the tau immunization study mentioned earlier as evidence in favor of PHF toxicity (115), it was found that immunization causes reduced aggregation of tau into PHFs or larger aggregates, and was associated with a reduced behavioral phenotype. However, this also causes an *increase* of PHF-1 immunoreactivity in the “high-speed soluble” fraction, which in this case would represent any species of GTO size or smaller. This suggests not only that PHFs might be toxic, but also that the soluble species present were not sufficient for toxicity (115).

The inducible pro-aggregation and anti-aggregation mutants of the tau repeat regions created by the Mandelkow laboratory argue against toxicity of soluble tau. In both the cell lines [the study mentioned as evidence against PHF toxicity: (54)], and in the mice (95, 98), the common theme was that the anti-aggregation mutant, which would always be a soluble form of tau, was never toxic. The mouse lines provide some additional correlative evidence against toxicity of any form of tau smaller than PHFs, in that the levels of “soluble” tau were constant between the pro-aggregation mouse, which experienced neurotoxicity, and the anti-aggregation mouse, which did not.

Clearly, physiological tau is soluble and non-toxic. However, under pathological conditions tau may undergo changes that render it toxic, even though it may remain soluble.

### Distinguishing between small (soluble) tau oligomers and tau monomers

In the above paragraphs, a general trend is that the evidence for soluble tau toxicity centers on some reasonably well-defined dimers and trimers which are demonstrably sufficient for toxicity; whereas the evidence against either has not distinguished between the myriad species, or has only said that one particular type of soluble tau is not toxic. In that sense, the evidence against is not conclusive. While the specific data cited in favor of dimer and trimer toxicity is compelling, we still know very little about the properties (or indeed existence) of all the other soluble forms.

## Conclusion

**Figure 2:**
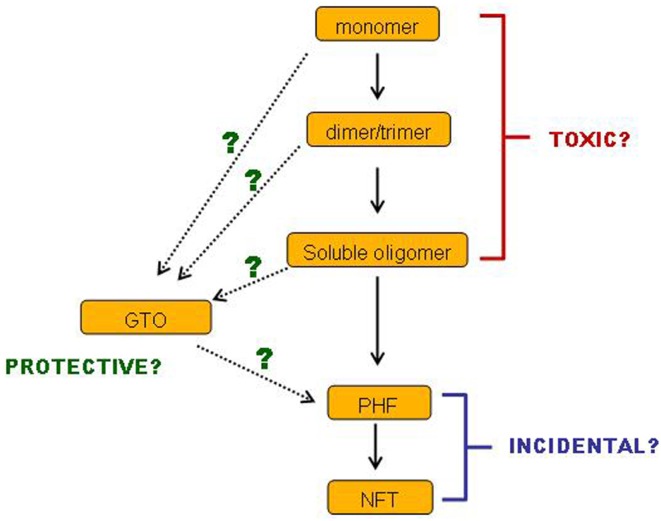
**Cartoon of remaining questions**.

In conclusion, there is a body of evidence demonstrating that small soluble tau oligomers are the most toxic form of tau. Filamentous and fibrillar tau is neither necessary nor sufficient for tau-induced toxicity, and may very well represent a neuroprotective strategy. Such ideas are not new, and a number of reviews over the past few years have drawn the same conclusions (138–140). Nevertheless, this conclusion is still not broadly accepted. Even if it were to be accepted, many questions remain. We still have little idea which of the multiplicity of soluble tau species is the culprit or culprits: is it monomers, dimers or trimers or all three, and in which conformation(s) and phosphorylation state(s)? Also, we have incomplete information about the sequence of events on the pathway(s) of tau aggregation (Figure 2). Especially, where do GTOs fit in? Are they made gradually from increasing sizes of smaller oligomers? Or do they have a different conformation that makes them so compact and insoluble? Can they really go on to form PHFs *in vivo*? Such questions are important for informing the strategies to be implemented when developing treatments for AD and tauopathies. We do not yet know which species of tau would represent the best target for tau-based therapies. If a certain specific set of small soluble tau oligomers are toxic, while insoluble GTOs and larger insoluble tau species are not, then perhaps strategies aimed at breaking up large insoluble tau aggregates might prove ineffective. Especially if it transpires that GTOs are not only non-toxic but on an alternative pathway to PHFs, then perhaps *encouraging* GTO formation might even turn out to be a valid approach. Alternatively, it might not be size and solubility alone of the tau species that are the key toxicity-determining factors: levels of β-sheet structure and phosphorylation at certain sites may also be influential.

There is a precedent from other proteinopathies for a small soluble species being the most toxic, while the smallest insoluble form is relatively protective. This has been demonstrated for huntingtin protein in Hungtington’s disease (141, 142), alpha-synuclein in Parkinson’s disease (143), and amyloid beta in AD (144): however, evidence for this phenomenon in the case of tau aggregation is only beginning to emerge now.

## Conflict of Interest Statement

The authors declare that the research was conducted in the absence of any commercial or financial relationships that could be construed as a potential conflict of interest.

## Footnotes

^1^ For reference, stage 1 in this system is filamentous silver-stained tangles composed primarily of PHF; stage 2 is a classic neurofibrillary tangle and stage 3 is a ghost tangle (See “Neurofibrillary Tangle” and “Ghost Tangle”
